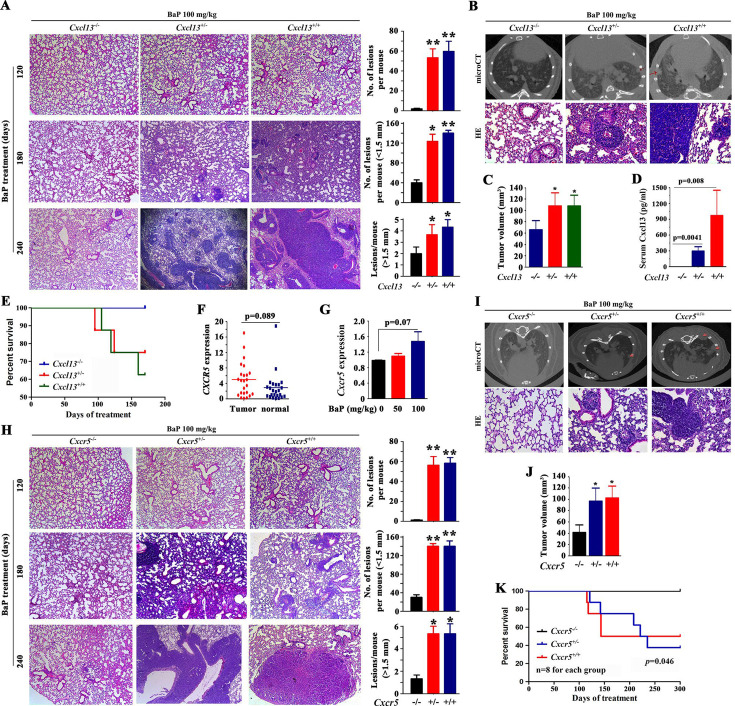# Correction: The chemokine CXCL13 in lung cancers associated with environmental polycyclic aromatic hydrocarbons pollution

**DOI:** 10.7554/eLife.112818

**Published:** 2026-08-06

**Authors:** Gui-Zhen Wang, Xin Cheng, Bo Zhou, Zhe-Sheng Wen, Yun-Chao Huang, Hao-Bin Chen, Gao-Feng Li, Zhi-Liang Huang, Yong-Chun Zhou, Lin Feng, Ming-Ming Wei, Li-Wei Qu, Yi Cao, Guang-Biao Zhou

**Keywords:** Human

Wang G-Z, Cheng X, Zhou B, Wen Z-S, Huang Y-C, Chen H-B, Li G-F, Huang Z-L, Zhou Y-C, Feng L, Wei M-M, Qu L-W, Cao Y, Zhou G-B. 2015. The chemokine CXCL13 in lung cancers associated with environmental polycyclic aromatic hydrocarbons pollution. eLife **4**:e09419. doi: 10.7554/eLife.09419.

Published 13 November 2015

This correction addresses two errors we identified in the published article.

1) In Figure 2C, images from the time-course experiment (0–72 h) were inadvertently copied and pasted into the concentration-response panel (0–10 µM) of the bar charts for 16HBE and A549 cells treated with BaP, resulting in image duplication; however, the *p*-value annotations correctly correspond to the concentration data. Additionally, the labels for the brown and green bars in the time-course panel were accidentally swapped: the brown bar represents 72 h and the green bar represents 48 h.

2) Figure 4A shows H&E staining of lung tissue from mice of different genotypes treated with 100 mg/kg BaP by gavage for 120, 180, and 240 days. During subsequent review, we found that the images for the 120 day treatment group had been inadvertently misarranged during figure assembly. Specifically, because *Cxcl13*⁻^/^⁻ and *Cxcr5*⁻^/^⁻ mice showed no lung abnormalities after 120 days of BaP treatment, whereas *Cxcl13*^+/^⁻ and *Cxcl13*^+/+^ mice exhibited similar lung injury at this time point, the high morphological similarity of the H&E-stained sections led to accidental misplacement of images between genotypes, resulting in overlapping fields of view among images used for different experimental conditions. These errors have now been corrected by replacing the panels with the correct images, which were retrieved from the original photographic records.

We highlight these overlaps below using blue or red boxes (i) within Figure 4A (*Cxcl13*^+/-^ and *Cxcl13*^+/+^) and (ii) between Figure 4A/4 H (*Cxcl1*3^-/-^ and *Cxcr5*^-/-^):

**Figure 1:**
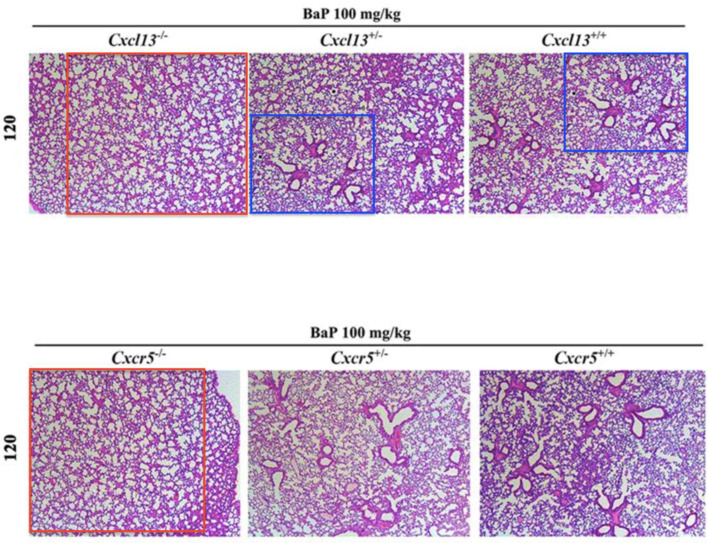


The corrected Figure 2 (updated for panel C) is shown below:

**Figure 2:**
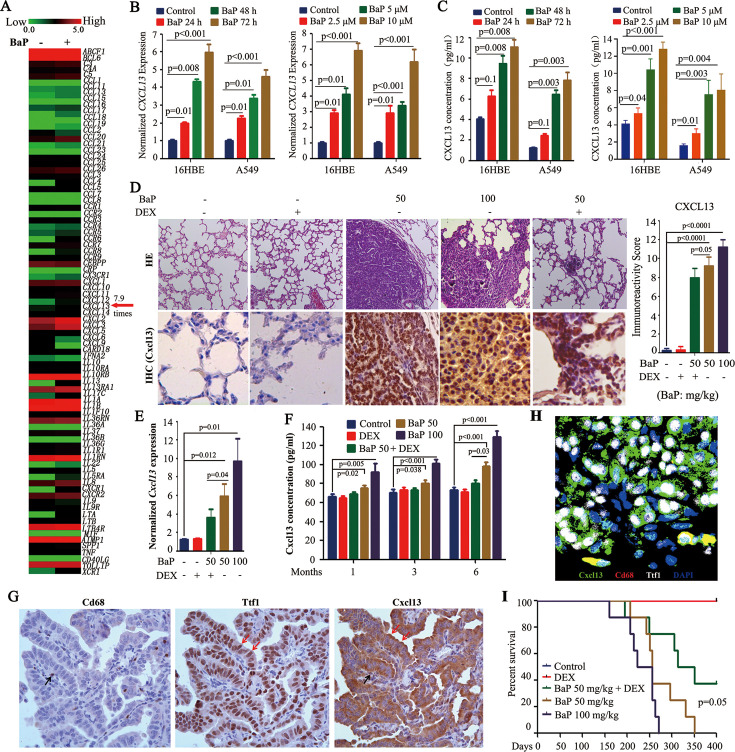


The originally published Figure 2 is shown for reference:

**Figure 3:**
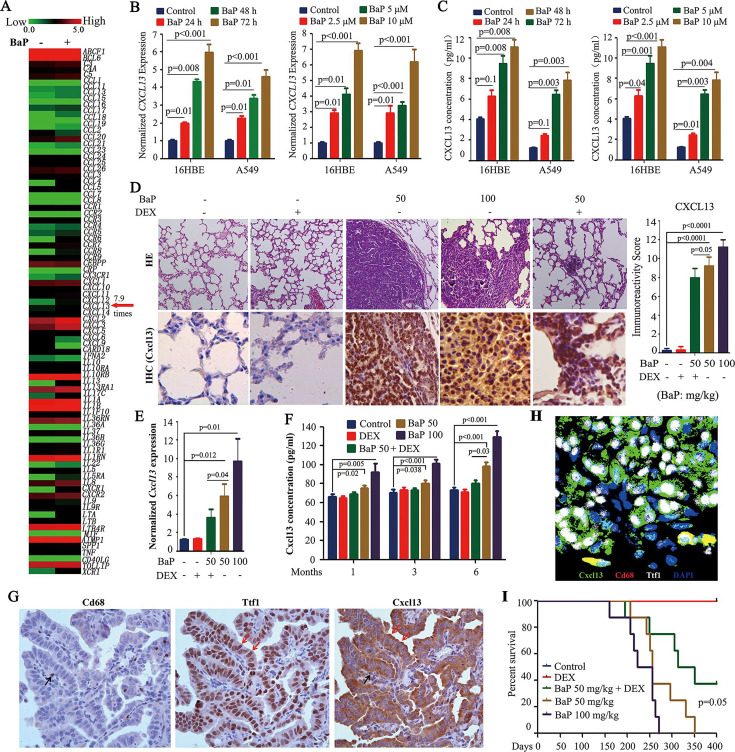


The corrected Figure 4 (updated for panel A) is shown below:

**Figure 4:**
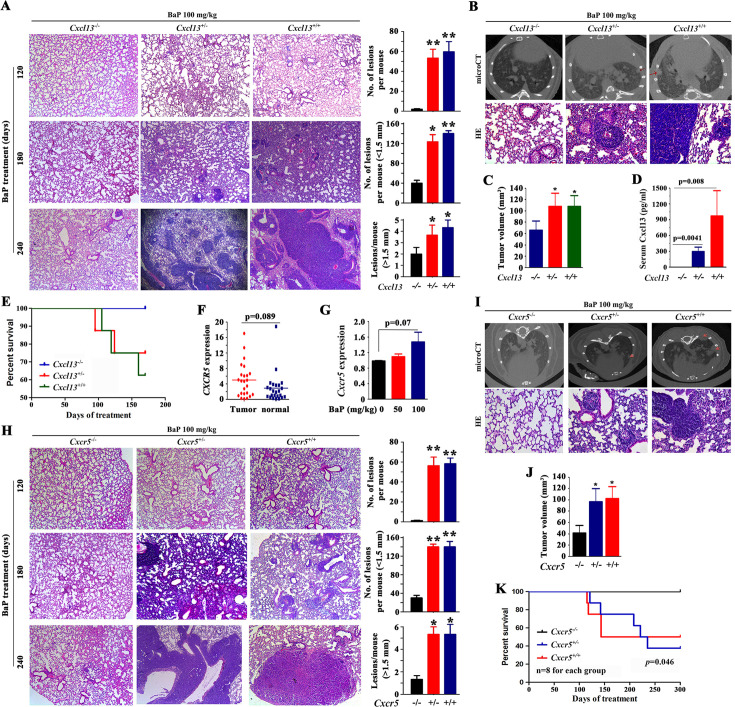


The originally published Figure 4 is shown for reference:

**Figure 5:**