# Supplementary material for: The chemokine CXCL13 in lung cancers associated with environmental polycyclic aromatic hydrocarbons pollution
Source: eLife. 2015 Nov 13;4:e09419. doi: 10.7554/eLife.09419 (PMC4764582; doi:10.7554/eLife.09419)
Supplement: Figure 1—source data 1. — DOI: http://dx.doi.org/10.7554/eLife.09419.004 [file elife-09419-fig1-data1.docx]

**Source data file 2. Sequences of primers for real-time PCR and ChIP, and siRNA**

| **Target** | **Forward primer (5’→3’)** | **Reverse primer (5’→3’)** |
| --- | --- | --- |
| **HUMAN** |  |  |
| *GAPDH* | GAAGGTGAAGGTCGGAGTC | GAAGATGGTGATGGGATTTC |
| *CXCL13* | CAGTCCAAGGTGTTCTGG | CAATGAAGCGTCTAGGGATAAAG |
| *CXCL2* | CAGACCTCCTGAGCCCAA | TGCAATCCCCCATCCTTTC |
| *CXCL11* | TGCTCACGTTCACCACC | TCTGCCACTTTCACTGCT |
| *CXCL12* | CGACTTGAGGCTGTGAGG | GGTGAACCCGAGAAGAGAC |
| *CXCL14* | TGTCCCTGCTCCCACGC | AGCTCCCCGCACTCACC |
| *SPP1* | TCGCAGACCTGACATCCAGTACC | CCATTCAACTCCTCGCTTTCCAT |
| *E-cadherin* | TGCCCAGAAAATGAAAAAGG | GTGTATGTGGCAATGCGTTC |
| *N-cadherin* | ACAGTGGCCACCTACAAAGG | CCGAGATGGGGTTGATAATG |
| *Vimentin* | GAGAACTTTGCCGTTGAAGC | GCTTCCTGTAGGTGGCAATC |
| *Slug* | GGGGAGAAGCCTTTTTCTTG | TCCTCATGTTTGTGCAGGAG |
| *Snail* | CCTCCCTGTCAGATGAGGAC | CCAGGCTGAGGTATTCCTTG |
| *MMP7* | GTATGGGACATTCCTCTGATCC | CCAATGAATGAATGAATGGATG |
| *TCF* | ATCTGTGTCCCATGTCCCAG | CCAGGGTAGGAGACTTGCAG |
| *MMP12* | GACAGGTTCTTCTGGTGGCTGGT | TGGCAAGGTTGGCCATAAG |
| *AhR* | AGGCTAGCCAAACGGTCCAAC | ATCACCTACGCCAGTCGCAAG |
| *C4orf7* | AGTGGCTGTTGGTTTCCCAG | GGCGAAATGGATATGGGTAAGG |
| *C9orf46* | TGCAGAGTGAAATGAGGGAA | AACAATCGGGACCAGGAA |
| **Mouse** |  |  |
| *Gaphd* | AGTATGACTCCACTCACGGCAA | TCTCGCTCCTGGAAGATGGT |
| *Cxcl13* | AACTCCACCTCCAGGCAGAATG | TGTGTAATGGGCTTCCAGAATACC |
| *Cxcr5* | GACTCCTTACCACAGTGCACCTT | GGAAACGGGAGGTGAACCA |
| *E-cadherin* | TCCTTGTTCGGCTATGTGTC | GGCATGCACCTAAGAATCAG |
| *N-cadherin* | AGTCACAGTGGTAAAACCAATTGA | GGCTGTCGACTGAGGTGGGTG |
| *vimentin* | CCTTGAACGGAAAGTGGAATC | GACATGCTGTTCCTGAATCTGGG |
| **ChIP** |  |  |
| *CXCL13* | ATTTACACTCCCACCAACAG | GAGAATCCTGTGAACCCAG |
| **siRNA** | **Sequence 1 (5’→3’)** | **Sequence 2 (5’→3’)** |
| siAhR | GGAUUAAAUUAGUUUGUGAdTdT | UCACAAACUAAUUUAAUCCdAdA |
