# Supplementary material for: The chemokine CXCL13 in lung cancers associated with environmental polycyclic aromatic hydrocarbons pollution
Source: eLife. 2015 Nov 13;4:e09419. doi: 10.7554/eLife.09419 (PMC4764582; doi:10.7554/eLife.09419)
Supplement: Figure 6—source data 1. — DOI: http://dx.doi.org/10.7554/eLife.09419.017 [file elife-09419-fig6-data1.docx]

**Figure 6- Source data 1. *CXCL13*-associated genes in lung cancer**

| **No.** | **Gene Symbol** | **Gene Title** | **Mean CXCL13  Correlation** |
| --- | --- | --- | --- |
| 1 | CXCL13 | chemokine (C-X-C motif) ligand 13 | 1 |
| 2 | RNASEH2A | ribonuclease H2, subunit A | 0.826215 |
| 3 | MMP12 | matrix metallopeptidase 12 (macrophage elastase) | 0.818184 |
| 4 | SPP1 | secreted phosphoprotein 1 | 0.800919 |
| 5 | SSR3 | signal sequence receptor, gamma (translocon-associated protein gamma) | 0.79825 |
| 6 | IGLL5 | immunoglobulin lambda-like polypeptide 5 | 0.749201 |
| 7 | LTB | lymphotoxin beta (TNF superfamily, member 3) | 0.736191 |
| 8 | CLSTN3 | calsyntenin 3 | 0.731517 |
| 9 | CD7 | CD7 molecule | 0.715534 |
| 10 | SPIB | Spi-B transcription factor (Spi-1/PU.1 related) | 0.708745 |
| 11 | MGC29506 | hypothetical protein MGC29506 | 0.701335 |
| 12 | MMP7 | matrix metallopeptidase 7 (matrilysin, uterine) | 0.691338 |
| 13 | CCNA2 | cyclin A2 | 0.688587 |
| 14 | CD38 | CD38 molecule | 0.687553 |
| 15 | AS3MT | arsenic (+3 oxidation state) methyltransferase | 0.684414 |
| 16 | CHRDL2 | chordin-like 2 | 0.680313 |
| 17 | CHMP4B | chromatin modifying protein 4B | 0.678314 |
| 18 | MAN2B1 | mannosidase, alpha, class 2B, member 1 | 0.675359 |
| 19 | CFB | complement factor B | 0.675245 |
| 20 | OIP5 | Opa interacting protein 5 | 0.673951 |
| 21 | TGIF2 | TGFB-induced factor homeobox 2 | 0.672771 |
| 22 | RPL39L | ribosomal protein L39-like | 0.671193 |
| 23 | PTPRCAP | protein tyrosine phosphatase, receptor type, C-associated protein | 0.670833 |
| 24 | MS4A1 | membrane-spanning 4-domains, subfamily A, member 1 | 0.666562 |
| 25 | CCDC99 | coiled-coil domain containing 99 | 0.665692 |
| 26 | 1-Sep | Serologically Defined Breast Cancer | 0.664137 |
| 27 | LOC100132247 | nuclear pore complex interacting protein-like 3; similar to Uncharacterized protein KIAA0220 | 0.663918 |
| 28 | CCL19 | chemokine (C-C motif) ligand 19 | 0.657868 |
| 29 | CD72 | CD72 molecule | 0.655089 |
| 30 | MAD2L1 | MAD2 mitotic arrest deficient-like 1 (yeast) | 0.651938 |
| 31 | TCF | transcription factor (E2A immunoglobulin enhancer binding factors E12/E47) | 0.649679 |
| 32 | C7orf68 | chromosome 7 open reading frame 68 | 0.648668 |
| 33 | MTL5 | metallothionein-like 5, testis-specific (tesmin) | 0.640864 |
| 34 | ITCH | itchy E3 ubiquitin protein ligase homolog (mouse) | 0.637669 |
| 35 | STAP1 | signal transducing adaptor family member 1 | 0.637502 |
| 36 | CP | ceruloplasmin (ferroxidase) | 0.635231 |
| 37 | QRSL1 | glutaminyl-tRNA synthase (glutamine-hydrolyzing)-like 1 | 0.633888 |
| 38 | AP1G1 | adaptor-related protein complex 1, gamma 1 subunit | 0.633691 |
| 39 | IGLL1 | immunoglobulin lambda-like polypeptide 1 | 0.632993 |
| 40 | PIP4K2A | phosphatidylinositol-5-phosphate 4-kinase, type II, alpha | 0.632278 |
| 41 | IDH2 | isocitrate dehydrogenase 2 (NADP+), mitochondrial | 0.631769 |
| 42 | YBX2 | Y box binding protein 2 | 0.626672 |
| 43 | MMP9 | matrix metallopeptidase 9 (gelatinase B, 92kDa gelatinase, 92kDa type IV collagenase) | 0.625831 |
| 44 | FNBP1 | formin binding protein 1 | 0.62408 |
| 45 | C9orf46 | chromosome 9 open reading frame 46 | 0.624039 |
| 46 | CORO1A | coronin, actin binding protein, 1A | 0.623735 |
| 47 | CXCL10 | chemokine (C-X-C motif) ligand 10 | 0.62133 |
| 48 | HP | haptoglobin-related protein; haptoglobin | 0.620195 |
| 49 | SAA1 | serum amyloid A1 | 0.616561 |
| 50 | C4orf7 | chromosome 4 open reading frame 7 | 0.613882 |
| 51 | HSH2D | hematopoietic SH2 domain containing | 0.611848 |
| 52 | CYP7B1 | cytochrome P450, family 7, subfamily B, polypeptide 1 | 0.611082 |
| 53 | CKM | creatine kinase, muscle | 0.609143 |
| 54 | PNOC | prepronociceptin | 0.608669 |
| 55 | CTHRC1 | collagen triple helix repeat containing 1 | 0.608272 |
| 56 | ZDHHC3 | zinc finger, DHHC-type containing 3 | 0.607517 |
| 57 | LSG1 | large subunit GTPase 1 homolog (S. cerevisiae) | 0.607419 |
| 58 | CD8B | CD8b molecule | 0.607008 |
| 59 | C2CD4A | family with sequence similarity 148, member A | 0.604457 |
| 60 | LRRC8B | leucine rich repeat containing 8 family, member B | 0.602435 |
| 61 | TTYH3 | tweety homolog 3 (Drosophila) | 0.599532 |
| 62 | CDK1 | cell division cycle 2, G1 to S and G2 to M | 0.598167 |
| 63 | CD27 | CD27 molecule | 0.594933 |
| 64 | HNRNPAB | heterogeneous nuclear ribonucleoprotein A/B | 0.594708 |
| 65 | ATF5 | activating transcription factor 5 | 0.594174 |
| 66 | HLA-DOB | major histocompatibility complex, class II, DO beta | 0.593583 |
| 67 | HEATR3 | HEAT repeat containing 3 | 0.592324 |
| 68 | SPEN | spen homolog, transcriptional regulator (Drosophila) | 0.592312 |
| 69 | GPX7 | glutathione peroxidase 7 | 0.591868 |
| 70 | FAM104A | family with sequence similarity 104, member A | 0.591518 |
| 71 | ZCCHC17 | zinc finger, CCHC domain containing 17 | 0.591396 |
| 72 | SUV39H2 | suppressor of variegation 3-9 homolog 2 (Drosophila) | 0.590719 |
| 73 | UBE2C | ubiquitin-conjugating enzyme E2C | 0.589869 |
| 74 | TYMS | thymidylate synthetase | 0.588333 |
| 75 | COG5 | component of oligomeric golgi complex 5 | 0.587825 |
| 76 | RIPK2 | receptor-interacting serine-threonine kinase 2 | 0.58701 |
| 77 | SEC61G | Sec61 gamma subunit | 0.586611 |
| 78 | DFFA | DNA fragmentation factor, 45kDa, alpha polypeptide | 0.585147 |
| 79 | HJURP | Holliday junction recognition protein | 0.584591 |
| 80 | GPC2 | glypican 2 | 0.583355 |
| 81 | STAT5B | signal transducer and activator of transcription 5B | 0.582355 |
| 82 | CCAR1 | cell division cycle and apoptosis regulator 1 | 0.582034 |
| 83 | ADAP2 | ArfGAP with dual PH domains 2 | 0.580614 |
| 84 | KIAA0556 | KIAA0556 | 0.580448 |
| 85 | TMEM149 | transmembrane protein 149 | 0.580354 |
| 86 | GAPDH | glyceraldehyde-3-phosphate dehydrogenase-like 6 | 0.579183 |
| 87 | HM13 | histocompatibility (minor) 13 | 0.576076 |
| 88 | DCBLD2 | discoidin, CUB and LCCL domain containing 2 | 0.573019 |
| 89 | CHEK2 | protein kinase CHK2-like; CHK2 checkpoint homolog (S. pombe); similar to hCG1983233 | 0.572387 |
| 90 | TTC9C | tetratricopeptide repeat domain 9C | 0.568041 |
| 91 | KIAA0430 | KIAA0430 | 0.566067 |
| 92 | 6-Sep | KIAA0128 | 0.565602 |
| 93 | PAK1IP1 | PAK1 interacting protein 1 | 0.565369 |
| 94 | CD2 | CD2 molecule | 0.564184 |
| 95 | NCAPD2 | non-SMC condensin I complex, subunit D2 | 0.563898 |
| 96 | IGF1 | insulin-like growth factor 1 (somatomedin C) | 0.562316 |
| 97 | ADCK2 | aarF domain containing kinase 2 | 0.561998 |
| 98 | ZDHHC21 | zinc finger, DHHC-type containing 21 | 0.559903 |
| 99 | RALGPS2 | Ral GEF with PH domain and SH3 binding motif 2 | 0.559631 |
| 100 | AASS | aminoadipate-semialdehyde synthase | 0.55962 |
| 101 | NAPG | N-ethylmaleimide-sensitive factor attachment protein, gamma | 0.557821 |
| 102 | CCR7 | chemokine (C-C motif) receptor 7 | 0.556274 |
| 103 | EIF2C3 | eukaryotic translation initiation factor 2C, 3 | 0.556227 |
| 104 | BRAF | v-raf murine sarcoma viral oncogene homolog B1 | 0.555955 |
| 105 | UTP6 | UTP6, small subunit (SSU) processome component, homolog (yeast) | 0.555788 |
| 106 | MBTD1 | mbt domain containing 1 | 0.555432 |
| 107 | METTL4 | methyltransferase like 4 | 0.554663 |
| 108 | C7orf44 | chromosome 7 open reading frame 44 | 0.552787 |
| 109 | HCP5 | HLA complex P5 | 0.552636 |
| 110 | GGCT | gamma-glutamyl cyclotransferase | 0.552501 |
| 111 | RAB42 | RAB42, member RAS oncogene family | 0.552029 |
| 112 | PPP1R14B | protein phosphatase 1, regulatory (inhibitor) subunit 14B | 0.55068 |
| 113 | SPC25 | SPC25, NDC80 kinetochore complex component, homolog (S. cerevisiae) | 0.549389 |
| 114 | NOM1 | nucleolar protein with MIF4G domain 1 | 0.548872 |
| 115 | CYSLTR1 | cysteinyl leukotriene receptor 1 | 0.548532 |
| 116 | BARD1 | BRCA1 associated RING domain 1 | 0.548457 |
| 117 | RPL7L1 | ribosomal protein L7-like 1; ribosomal protein L7 pseudogene 14 | 0.547741 |
| 118 | WDYHV1 | WDYHV motif containing 1 | 0.547117 |
| 119 | C1orf51 | chromosome 1 open reading frame 51 | 0.546896 |
| 120 | MRPS25 | mitochondrial ribosomal protein S25 | 0.546801 |
| 121 | APOE | hypothetical LOC100129500; apolipoprotein E | 0.545215 |
| 122 | SH3KBP1 | SH3-domain kinase binding protein 1 | 0.544764 |
| 123 | DDX55 | DEAD (Asp-Glu-Ala-Asp) box polypeptide 55 | 0.544386 |
| 124 | CENPH | centromere protein H | 0.544132 |
| 125 | RUNX1 | runt-related transcription factor 1 | 0.543596 |
| 126 | EPHA4 | EPH receptor A4 | 0.542767 |
| 127 | CUEDC1 | CUE domain containing 1 | 0.541677 |
| 128 | CPSF6 | cleavage and polyadenylation specific factor 6, 68kDa | 0.541618 |
| 129 | P2RX5 | purinergic receptor P2X, ligand-gated ion channel, 5 | 0.541595 |
| 130 | WDR86 | WD repeat domain 86 | 0.541327 |
| 131 | P2RY8 | purinergic receptor P2Y, G-protein coupled, 8 | 0.541022 |
| 132 | HIVEP1 | human immunodeficiency virus type I enhancer binding protein 1 | 0.540804 |
| 133 | KIAA0406 | KIAA0406 | 0.54063 |
| 134 | POSTN | periostin, osteoblast specific factor | 0.539387 |
| 135 | SFRS14 | splicing factor, arginine/serine-rich 14 | 0.539038 |
| 136 | ZNF451 | zinc finger protein 451 | 0.538472 |
| 137 | ARID5B | AT rich interactive domain 5B (MRF1-like) | 0.538092 |
| 138 | GGA3 | golgi associated, gamma adaptin ear containing, ARF binding protein 3 | 0.537033 |
| 139 | PTP4A2 | protein tyrosine phosphatase type IVA, member 2 | 0.536797 |
| 140 | ERO1L | ERO1-like (S. cerevisiae) | 0.536504 |
| 141 | EIF2B2 | eukaryotic translation initiation factor 2B, subunit 2 beta, 39kDa | 0.536232 |
| 142 | STEAP1 | six transmembrane epithelial antigen of the prostate 1 | 0.535751 |
| 143 | CCDC93 | coiled-coil domain containing 93 | 0.534227 |
| 144 | LOC729020 | rcRPE; ribulose-5-phosphate-3-epimerase | 0.534084 |
| 145 | RASSF1 | Ras association (RalGDS/AF-6) domain family member 1 | 0.532434 |
| 146 | ZNF250 | zinc finger protein 250 | 0.531216 |
| 147 | SNF8 | SNF8, ESCRT-II complex subunit, homolog (S. cerevisiae) | 0.530805 |
| 148 | CYP1B1 | cytochrome P450, family 1, subfamily B, polypeptide 1 | 0.530697 |
| 149 | BTN3A1 | butyrophilin, subfamily 3, member A1 | 0.5293 |
| 150 | CNOT1 | CCR4-NOT transcription complex, subunit 1 | 0.529037 |
| 151 | C4B | complement component 4B (Chido blood group) | 0.527834 |
| 152 | CENPA | centromere protein A | 0.527045 |
| 153 | VRK1 | vaccinia related kinase 1 | 0.526568 |
| 154 | PTTG2 | pituitary tumor-transforming 1; pituitary tumor-transforming 2 | 0.526113 |
| 155 | SLAMF1 | signaling lymphocytic activation molecule family member 1 | 0.526065 |
| 156 | PDK1 | pyruvate dehydrogenase kinase, isozyme 1 | 0.525664 |
| 157 | C3orf62 | chromosome 3 open reading frame 62 | 0.525648 |
| 158 | ZNF720 | zinc finger protein 720 | 0.525587 |
| 159 | CHD6 | chromodomain helicase DNA binding protein 6 | 0.523777 |
| 160 | SPAG4 | sperm associated antigen 4 | 0.522781 |
| 161 | TMEM106A | hypothetical LOC728772; transmembrane protein 106A | 0.522663 |
| 162 | NUSAP1 | nucleolar and spindle associated protein 1 | 0.522156 |
| 163 | C15orf63 | chromosome 15 open reading frame 63; small EDRK-rich factor 2 | 0.52111 |
| 164 | C1orf63 | chromosome 1 open reading frame 63 | 0.520596 |
| 165 | USP45 | ubiquitin specific peptidase 45 | 0.520212 |
| 166 | OSTalpha | organic solute transporter alpha | 0.520036 |
| 167 | DDX46 | DEAD (Asp-Glu-Ala-Asp) box polypeptide 46 | 0.519166 |
| 168 | SDCCAG3 | serologically defined colon cancer antigen 3 | 0.518887 |
| 169 | ICAM3 | intercellular adhesion molecule 3 | 0.518511 |
| 170 | PSMD1 | proteasome (prosome, macropain) 26S subunit, non-ATPase, 1 | 0.518379 |
| 171 | LOC649330 | heterogeneous nuclear ribonucleoprotein C-like | 0.517675 |
| 172 | TNFRSF18 | tumor necrosis factor receptor superfamily, member 18 | 0.51713 |
| 173 | C3orf14 | chromosome 3 open reading frame 14 | 0.515819 |
| 174 | PTTG1 | pituitary tumor-transforming 1; pituitary tumor-transforming 2 | 0.514054 |
| 175 | ARHGAP15 | Rho GTPase activating protein 15 | 0.513593 |
| 176 | RASSF2 | Ras association (RalGDS/AF-6) domain family member 2 | 0.513174 |
| 177 | SCTR | secretin receptor | 0.512281 |
| 178 | MAPK13 | mitogen-activated protein kinase 13 | 0.512273 |
| 179 | GRB2 | growth factor receptor-bound protein 2 | 0.512213 |
| 180 | SMN1 | survival of motor neuron 1, telomeric; survival of motor neuron 2, centromeric | 0.512085 |
| 181 | RPL26L1 | ribosomal protein L26-like 1 | 0.511982 |
| 182 | ATIC | 5-aminoimidazole-4-carboxamide ribonucleotide formyltransferase/IMP cyclohydrolase | 0.511932 |
| 183 | SART3 | squamous cell carcinoma antigen recognized by T cells 3 | 0.511754 |
| 184 | ZCCHC7 | zinc finger, CCHC domain containing 7 | 0.511696 |
| 185 | EIF1AY | eukaryotic translation initiation factor 1A, Y-linked | 0.51169 |
| 186 | DSN1 | DSN1, MIND kinetochore complex component, homolog (S. cerevisiae) | 0.511239 |
| 187 | CALML5 | calmodulin-like 5 | 0.51033 |
| 188 | RFT1 | RFT1 homolog (S. cerevisiae) | 0.509955 |
| 189 | RPL39 | ribosomal protein L39 pseudogene 10; ribosomal protein L39 pseudogene 20 | 0.509864 |
| 190 | GMPPB | GDP-mannose pyrophosphorylase B | 0.509516 |
| 191 | ABI2 | abl interactor 2 | 0.509154 |
| 192 | SAMD4B | sterile alpha motif domain containing 4B | 0.508602 |
| 193 | PSME3 | proteasome (prosome, macropain) activator subunit 3 (PA28 gamma; Ki) | 0.508521 |
| 194 | BHLHE22 | basic helix-loop-helix family, member e22 | 0.508406 |
| 195 | DAZAP1 | DAZ associated protein 1 | 0.508312 |
| 196 | PHKG2 | phosphorylase kinase, gamma 2 (testis) | 0.507531 |
| 197 | RAD1 | RAD1 homolog (S. pombe) | 0.506521 |
| 198 | SP9 | solute carrier family 29 (nucleoside transporters) | 0.50562 |
| 199 | MAL2 | mal, T-cell differentiation protein 2 | 0.505158 |
| 200 | BTN3A2 | butyrophilin, subfamily 3, member A2 | 0.503416 |
| 201 | FAM156B | family with sequence similarity 156, member A; family with sequence similarity 156, member B | 0.503023 |
| 202 | CXCL9 | chemokine (C-X-C motif) ligand 9 | 0.502773 |
| 203 | CALU | calumenin | 0.502613 |
| 204 | MTRR | 5-methyltetrahydrofolate-homocysteine methyltransferase reductase | 0.502373 |
| 205 | KIAA0101 | KIAA0101 | 0.502321 |
| 206 | SRRT | serrate RNA effector molecule homolog (Arabidopsis) | 0.501577 |
| 207 | RFXANK | regulatory factor X-associated ankyrin-containing protein | 0.501518 |
| 208 | RAB34 | RAB34, member RAS oncogene family | 0.501319 |
| 209 | KIF11 | kinesin family member 11 | 0.500802 |
| 210 | MFSD8 | major facilitator superfamily domain containing 8 | 0.500706 |
| 211 | DTNA | dystrobrevin, alpha | -0.500069 |
| 212 | HCFC1R1 | host cell factor C1 regulator 1 (XPO1 dependent) | -0.500183 |
| 213 | PAX6 | paired box 6 | -0.501029 |
| 214 | EMP2 | epithelial membrane protein 2 | -0.501228 |
| 215 | KHNYN | KIAA0323 | -0.501627 |
| 216 | POU4F2 | POU class 4 homeobox 2 | -0.501699 |
| 217 | GDNF | glial cell derived neurotrophic factor | -0.502181 |
| 218 | ADAM33 | ADAM metallopeptidase domain 33 | -0.502278 |
| 219 | TEX11 | testis expressed 11 | -0.502693 |
| 220 | OR1N1 | olfactory receptor, family 1, subfamily N, member 1 | -0.503754 |
| 221 | C20orf194 | chromosome 20 open reading frame 194 | -0.504273 |
| 222 | HINFP | histone H4 transcription factor | -0.50438 |
| 223 | PCDHA3 | protocadherin alpha 3 | -0.504554 |
| 224 | ATN1 | atrophin 1 | -0.505532 |
| 225 | DAZL | deleted in azoospermia-like | -0.505582 |
| 226 | DSEL | dermatan sulfate epimerase-like | -0.505848 |
| 227 | ZBTB39 | zinc finger and BTB domain containing 39 | -0.506129 |
| 228 | CALCR | calcitonin receptor | -0.506164 |
| 229 | WIBG | within bgcn homolog (Drosophila) | -0.506464 |
| 230 | CEBPG | CCAAT/enhancer binding protein (C/EBP), gamma | -0.506707 |
| 231 | FAM125B | family with sequence similarity 125, member B | -0.507264 |
| 232 | ARC | activity-regulated cytoskeleton-associated protein | -0.507631 |
| 233 | CLRN3 | clarin 3 | -0.509135 |
| 234 | C12orf72 | chromosome 12 open reading frame 72 | -0.509176 |
| 235 | OR51T1 | olfactory receptor, family 51, subfamily T, member 1 | -0.509557 |
| 236 | SIPA1 | signal-induced proliferation-associated 1 | -0.510126 |
| 237 | TTLL7 | tubulin tyrosine ligase-like family, member 7 | -0.510584 |
| 238 | C15orf52 | chromosome 15 open reading frame 52 | -0.512022 |
| 239 | LOC100287718 | similar to ankyrin repeat domain 2 | -0.512094 |
| 240 | HSD17B12 | hydroxysteroid (17-beta) dehydrogenase 12 | -0.51222 |
| 241 | UTP14C | UTP14, U3 small nucleolar ribonucleoprotein, homolog C | -0.512361 |
| 242 | PRKCSH | protein kinase C substrate 80K-H | -0.51274 |
| 243 | DEFA4 | defensin, alpha 4, corticostatin | -0.513323 |
| 244 | ZFPM1 | zinc finger protein, multitype 1 | -0.513796 |
| 245 | 10-Sep | sept1-like | -0.514002 |
| 246 | CST4 | cystatin S | -0.514471 |
| 247 | POC1A | WD repeat domain 51A | -0.514707 |
| 248 | FIBIN | fin bud initiation factor homolog (zebrafish) | -0.514744 |
| 249 | MRPL41 | mitochondrial ribosomal protein L41 | -0.515514 |
| 250 | KBTBD7 | kelch repeat and BTB (POZ) domain containing 7 | -0.5166 |
| 251 | C10orf71 | chromosome 10 open reading frame 71; similar to hCG1996658 | -0.516858 |
| 252 | ITPRIPL2 | inositol 1,4,5-triphosphate receptor interacting protein-like 2 | -0.516959 |
| 253 | IFT88 | intraflagellar transport 88 homolog (Chlamydomonas) | -0.517963 |
| 254 | BAHD1 | bromo adjacent homology domain containing 1 | -0.518212 |
| 255 | NM_002141 | Homeobox Protein Hox-1D | -0.51853 |
| 256 | THSD7B | thrombospondin, type I, domain containing 7B | -0.519082 |
| 257 | PGLYRP1 | peptidoglycan recognition protein 1 | -0.520416 |
| 258 | CTDSP1 | CTD (carboxy-terminal domain, RNA polymerase II, polypeptide A) small phosphatase 1 | -0.520511 |
| 259 | KIF26A | kinesin family member 26A | -0.520711 |
| 260 | PLA2G1B | phospholipase A2, group IB (pancreas) | -0.521406 |
| 261 | MASP1 | mannan-binding lectin serine peptidase 1 (C4/C2 activating component of Ra-reactive factor) | -0.52179 |
| 262 | NAV1 | neuron navigator 1 | -0.522155 |
| 263 | SOSTDC1 | sclerostin domain containing 1 | -0.522235 |
| 264 | KCTD9 | similar to Potassium channel tetramerisation domain containing 9 | -0.522523 |
| 265 | CHD8 | chromodomain helicase DNA binding protein 8 | -0.522544 |
| 266 | IGFBP6 | insulin-like growth factor binding protein 6 | -0.522589 |
| 267 | NPDC1 | neural proliferation, differentiation and control, 1 | -0.522818 |
| 268 | ITGB1BP3 | integrin beta 1 binding protein 3 | -0.523199 |
| 269 | MLLT4 | similar to Afadin (Protein AF-6); myeloid/lymphoid or mixed-lineage leukemia | -0.52335 |
| 270 | PLLP | plasma membrane proteolipid (plasmolipin) | -0.52345 |
| 271 | LPPR1 | plasticity related gene 3 | -0.524845 |
| 272 | TPPP | tubulin polymerization promoting protein | -0.52539 |
| 273 | LRP10 | low density lipoprotein receptor-related protein 10 | -0.525471 |
| 274 | HPD | 4-hydroxyphenylpyruvate dioxygenase | -0.526003 |
| 275 | FXYD3 | FXYD domain containing ion transport regulator 3 | -0.52603 |
| 276 | IL17RC | interleukin 17 receptor C | -0.526399 |
| 277 | PCDHB16 | protocadherin beta 16 | -0.526557 |
| 278 | LCMT2 | leucine carboxyl methyltransferase 2 | -0.527036 |
| 279 | WFDC1 | WAP four-disulfide core domain 1 | -0.527262 |
| 280 | DNAJC17 | DnaJ (Hsp40) homolog, subfamily C, member 17 | -0.52766 |
| 281 | MOB2 | HCCA2 protein | -0.527952 |
| 282 | FAM123B | family with sequence similarity 123B | -0.528171 |
| 283 | TAB1 | mitogen-activated protein kinase kinase kinase 7 interacting protein 1 | -0.528436 |
| 284 | GPR126 | G protein-coupled receptor 126 | -0.528482 |
| 285 | NDUFC1 | NADH dehydrogenase (ubiquinone) 1, subcomplex unknown, 1, 6kDa | -0.529831 |
| 286 | NPFF | neuropeptide FF-amide peptide precursor | -0.529926 |
| 287 | LINGO4 | leucine rich repeat and Ig domain containing 4 | -0.530007 |
| 288 | KRTAP10-2 | keratin associated protein 10-2 | -0.530126 |
| 289 | MECP2 | methyl CpG binding protein 2 (Rett syndrome) | -0.530748 |
| 290 | MAP7 | microtubule-associated protein 7 | -0.53105 |
| 291 | FAM166B | family with sequence similarity 166, member B | -0.531874 |
| 292 | ITPKB | inositol 1,4,5-trisphosphate 3-kinase B | -0.532286 |
| 293 | PICK1 | protein interacting with PRKCA 1 | -0.532397 |
| 294 | PGRMC2 | progesterone receptor membrane component 2 | -0.533515 |
| 295 | AGPAT1 | 1-acylglycerol-3-phosphate O-acyltransferase 1 (lysophosphatidic acid acyltransferase, alpha) | -0.535774 |
| 296 | ANO6 | anoctamin 6 | -0.536219 |
| 297 | DOK7 | docking protein 7 | -0.536528 |
| 298 | RNMTL1 | RNA methyltransferase like 1 | -0.537595 |
| 299 | CAMK2B | calcium/calmodulin-dependent protein kinase II beta | -0.537747 |
| 300 | PCDHGA8 | protocadherin gamma subfamily A, 8 | -0.537775 |
| 301 | SOCS5 | suppressor of cytokine signaling 5 | -0.538609 |
| 302 | LENG9 | leukocyte receptor cluster (LRC) member 9 | -0.538931 |
| 303 | WISP2 | WNT1 inducible signaling pathway protein 2 | -0.539372 |
| 304 | TTLL8 | tubulin tyrosine ligase-like family, member 8 | -0.539737 |
| 305 | EFNB1 | ephrin-B1 | -0.540231 |
| 306 | TEAD4 | TEA domain family member 4 | -0.540245 |
| 307 | GRM1 | glutamate receptor, metabotropic 1 | -0.540814 |
| 308 | FAM69B | family with sequence similarity 69, member B | -0.541484 |
| 309 | EDA2R | ectodysplasin A2 receptor | -0.54232 |
| 310 | PAX9 | paired box 9 | -0.542552 |
| 311 | FLYWCH1 | FLYWCH-type zinc finger 1 | -0.543527 |
| 312 | ZBTB16 | zinc finger and BTB domain containing 16 | -0.54355 |
| 313 | TP53AIP1 | tumor protein p53 regulated apoptosis inducing protein 1 | -0.544393 |
| 314 | PPP1R11 | protein phosphatase 1, regulatory (inhibitor) subunit 11 | -0.544809 |
| 315 | NRCAM | neuronal cell adhesion molecule | -0.544856 |
| 316 | HBZ | hemoglobin, zeta | -0.54486 |
| 317 | ZBTB7C | zinc finger and BTB domain containing 7C | -0.54564 |
| 318 | HOXA2 | homeobox A2 | -0.54582 |
| 319 | SGCD | sarcoglycan, delta (35kDa dystrophin-associated glycoprotein) | -0.546107 |
| 320 | LILRA3 | leukocyte immunoglobulin-like receptor, subfamily A (without TM domain), member 3 | -0.546244 |
| 321 | ITPK1 | inositol 1,3,4-triphosphate 5/6 kinase | -0.54677 |
| 322 | PCDHA11 | protocadherin alpha 11 | -0.547215 |
| 323 | IL5RA | interleukin 5 receptor, alpha | -0.548136 |
| 324 | HAVCR1 | hepatitis A virus cellular receptor 1 | -0.548285 |
| 325 | BSDC1 | BSD domain containing 1 | -0.548902 |
| 326 | GLP1R | glucagon-like peptide 1 receptor | -0.550619 |
| 327 | MGST3 | microsomal glutathione S-transferase 3 | -0.550788 |
| 328 | TMEM203 | transmembrane protein 203 | -0.550954 |
| 329 | LETMD1 | LETM1 domain containing 1 | -0.551384 |
| 330 | CDH24 | cadherin-like 24 | -0.55286 |
| 331 | FRAT2 | frequently rearranged in advanced T-cell lymphomas 2 | -0.553252 |
| 332 | MX1 | myxovirus (influenza virus) resistance 1, interferon-inducible protein p78 (mouse) | -0.553385 |
| 333 | PLCD3 | phospholipase C, delta 3 | -0.554204 |
| 334 | C1orf190 | chromosome 1 open reading frame 190 | -0.554375 |
| 335 | PACS2 | phosphofurin acidic cluster sorting protein 2 | -0.554907 |
| 336 | PCDHGB7 | protocadherin gamma subfamily B, 7 | -0.555589 |
| 337 | GPM6A | glycoprotein M6A | -0.555725 |
| 338 | CYP4F22 | cytochrome P450, family 4, subfamily F, polypeptide 22 | -0.556088 |
| 339 | WNT11 | wingless-type MMTV integration site family, member 11 | -0.556244 |
| 340 | PODXL | podocalyxin-like | -0.556275 |
| 341 | CHAC1 | ChaC, cation transport regulator homolog 1 (E. coli) | -0.556719 |
| 342 | DMTF1 | cyclin D binding myb-like transcription factor 1 | -0.557054 |
| 343 | AGPHD1 | aminoglycoside phosphotransferase domain containing 1 | -0.557757 |
| 344 | ARHGAP5 | Rho GTPase activating protein 5 | -0.557972 |
| 345 | PHLDB2 | pleckstrin homology-like domain, family B, member 2 | -0.558058 |
| 346 | EDARADD | EDAR-associated death domain | -0.558491 |
| 347 | HNRNPUL1 | heterogeneous nuclear ribonucleoprotein U-like 1 | -0.558859 |
| 348 | PPP2R2B | protein phosphatase 2 (formerly 2A), regulatory subunit B, beta isoform | -0.559736 |
| 349 | C22orf25 | chromosome 22 open reading frame 25 | -0.560428 |
| 350 | ENDOU | 26 serine protease | -0.5619 |
| 351 | PRKCD | protein kinase C, delta | -0.562435 |
| 352 | RUNX3 | runt-related transcription factor 3 | -0.562953 |
| 353 | PHF17 | PHD finger protein 17 | -0.563145 |
| 354 | FZD1 | frizzled homolog 1 (Drosophila) | -0.563364 |
| 355 | FIZ1 | FLT3-interacting zinc finger 1 | -0.56363 |
| 356 | IRS2 | insulin receptor substrate 2 | -0.564254 |
| 357 | UGT1A1 | UDP glucuronosyltransferase 1 family, polypeptide A3 | -0.565161 |
| 358 | CABP1 | calcium binding protein 1 | -0.565232 |
| 359 | GTF2H2D | general transcription factor IIH, polypeptide 2, 44kDa | -0.567559 |
| 360 | NM_006735 | Homeobox Protein Hox-1K | -0.568824 |
| 361 | DKK3 | dickkopf homolog 3 (Xenopus laevis) | -0.569273 |
| 362 | TOLLIP | toll interacting protein | -0.569673 |
| 363 | RHOU | ras homolog gene family, member U | -0.570936 |
| 364 | HRC | histidine rich calcium binding protein | -0.571865 |
| 365 | CYS1 | cystin 1 | -0.572064 |
| 366 | MORN4 | MORN repeat containing 4 | -0.572445 |
| 367 | MINK1 | misshapen-like kinase 1 (zebrafish) | -0.573064 |
| 368 | DGAT1 | diacylglycerol O-acyltransferase homolog 1 (mouse) | -0.574267 |
| 369 | ZNF358 | zinc finger protein 358 | -0.57435 |
| 370 | ACCSL | 1-aminocyclopropane-1-carboxylate synthase homolog (Arabidopsis)(non-functional)-like | -0.575339 |
| 371 | CCDC85C | coiled-coil domain containing 85C | -0.575826 |
| 372 | GATA2 | GATA binding protein 2 | -0.579912 |
| 373 | MSX1 | msh homeobox 1 | -0.580717 |
| 374 | MYOC | myocilin, trabecular meshwork inducible glucocorticoid response | -0.582226 |
| 375 | OLFM1 | olfactomedin 1 | -0.583237 |
| 376 | SPDYA | protein phosphatase 1, catalytic subunit, beta isoform; speedy homolog A (Xenopus laevis) | -0.584508 |
| 377 | IMP3 | IMP3, U3 small nucleolar ribonucleoprotein, homolog (yeast) | -0.586272 |
| 378 | CPT1A | carnitine palmitoyltransferase 1A (liver) | -0.587571 |
| 378 | WNT7A | wingless-type MMTV integration site family, member 7A | -0.587611 |
| 379 | TMIE | transmembrane inner ear | -0.587699 |
| 380 | ANKRD57 | ankyrin repeat domain 57 | -0.588342 |
| 381 | MEF2D | myocyte enhancer factor 2D | -0.588658 |
| 382 | F2RL3 | coagulation factor II (thrombin) receptor-like 3 | -0.58915 |
| 383 | C6orf136 | chromosome 6 open reading frame 136 | -0.590158 |
| 384 | CARD10 | caspase recruitment domain family, member 10 | -0.590257 |
| 385 | TRIM35 | tripartite motif-containing 35 | -0.590348 |
| 386 | C2 | complement component 2 | -0.590486 |
| 387 | NDUFA3 | NADH dehydrogenase (ubiquinone) 1 alpha subcomplex, 3, 9kDa | -0.591323 |
| 388 | OMD | osteomodulin | -0.591429 |
| 389 | SHROOM2 | shroom family member 2 | -0.592158 |
| 390 | IGFALS | insulin-like growth factor binding protein, acid labile subunit | -0.593846 |
| 391 | KEAP1 | kelch-like ECH-associated protein 1 | -0.594444 |
| 392 | S100A13 | S100 calcium binding protein A13 | -0.594451 |
| 393 | FOXL1 | forkhead box L1 | -0.594974 |
| 394 | CCDC120 | coiled-coil domain containing 120 | -0.59684 |
| 395 | PI16 | peptidase inhibitor 16 | -0.597101 |
| 396 | BEND7 | BEN domain containing 7 | -0.598162 |
| 397 | CACNG4 | calcium channel, voltage-dependent, gamma subunit 4 | -0.599138 |
| 398 | GNAZ | guanine nucleotide binding protein (G protein), alpha z polypeptide | -0.599156 |
| 399 | DGKA | diacylglycerol kinase, alpha 80kDa | -0.599623 |
| 400 | NCOA7 | nuclear receptor coactivator 7 | -0.600868 |
| 401 | SPHAR | S-phase response (cyclin related) | -0.601708 |
| 402 | FXC1 | fracture callus 1 homolog (rat) | -0.602561 |
| 403 | ZNF436 | zinc finger protein 436 | -0.602668 |
| 404 | VPS53 | vacuolar protein sorting 53 homolog (S. cerevisiae) | -0.605014 |
| 405 | NM_002147 | Homeobox Protein Hu-1 | -0.606727 |
| 406 | FAM36A | family with sequence similarity 36, member A | -0.607002 |
| 407 | IRF7 | interferon regulatory factor 7 | -0.614512 |
| 408 | C7orf41 | chromosome 7 open reading frame 41 | -0.615757 |
| 409 | LMX1B | LIM homeobox transcription factor 1, beta | -0.618327 |
| 410 | ZNF365 | zinc finger protein 365 | -0.618593 |
| 411 | SCARA3 | scavenger receptor class A, member 3 | -0.618604 |
| 412 | PUS1 | pseudouridylate synthase 1 | -0.625526 |
| 413 | ODF3 | outer dense fiber of sperm tails 3 | -0.626217 |
| 414 | CFHR3 | complement factor H-related 3 | -0.627378 |
| 415 | C2orf83 | chromosome 2 open reading frame 83 | -0.628157 |
| 416 | CHRM1 | cholinergic receptor, muscarinic 1 | -0.62874 |
| 417 | DDX52 | DEAD (Asp-Glu-Ala-Asp) box polypeptide 52 | -0.62924 |
| 418 | TBX5 | T-box 5 | -0.632348 |
| 419 | FAM174B | family with sequence similarity 174, member B | -0.633541 |
| 420 | NEDD4L | neural precursor cell expressed, developmentally down-regulated 4-like | -0.634508 |
| 421 | MYB | v-myb myeloblastosis viral oncogene homolog (avian) | -0.63521 |
| 422 | NM_018952 | Homeobox Protein Hu-2 | -0.640202 |
| 423 | TYSND1 | trypsin domain containing 1 | -0.640545 |
| 424 | TFCP2L1 | transcription factor CP2-like 1 | -0.645432 |
| 425 | SLC48A1 | solute carrier family 48 (heme transporter), member 1 | -0.647388 |
| 426 | RLIM | ring finger protein, LIM domain interacting; similar to ring finger protein (C3H2C3 type) 6 | -0.650152 |
| 427 | BMP6 | bone morphogenetic protein 6 | -0.65056 |
| 428 | FRAT1 | frequently rearranged in advanced T-cell lymphomas | -0.651777 |
| 429 | FBRSL1 | fibrosin-like 1 | -0.655212 |
| 430 | ZBTB45 | zinc finger and BTB domain containing 45 | -0.658189 |
| 431 | ALKBH5 | alkB, alkylation repair homolog 5 (E. coli) | -0.66476 |
| 432 | RGS9 | regulator of G-protein signaling 9 | -0.665336 |
| 433 | PCGF2 | polycomb group ring finger 2 | -0.670837 |
| 434 | FUT1 | fucosyltransferase 1 (galactoside 2-alpha-L-fucosyltransferase, H blood group) | -0.678935 |
| 435 | EDA | ectodysplasin A | -0.697857 |
| 436 | NM_004503 | Homeobox Protein Hox-3C | -0.722909 |
